# Feasibility of an Isometric Maximal Voluntary Contraction Test in Hematological Cancer Patients during Thrombocytopenia

**DOI:** 10.1155/2013/470489

**Published:** 2013-03-21

**Authors:** Philipp Zimmer, Freerk T. Baumann, Janis Ebel, Eva Maria Zopf, Wilhelm Bloch, Thomas Elter

**Affiliations:** ^1^Department of Molecular and Cellular Sport Medicine, German Sport University Cologne, Am Sportpark Müngersdorf 6, 50933 Köln, Germany; ^2^Department I of Internal Medicine, Center for Integrated Oncology Köln Bonn, University of Cologne, Kerpener Straße 62, 50937 Köln, Germany

## Abstract

*Introduction*. Resistance training is rarely offered to hemato-oncological patients in the daily clinical routine due to its potential harmful impact on the cardiovascular system and the long periods of thrombocytopenia experienced by these patients. Therefore, it is important to determine a valid assessment to define and control resistance training. In this study, the feasibility of a maximal voluntary contraction (MVC) test was investigated in hemato-oncological patients. This inexpensive assessment may be a practicable alternative to the one repetition maximum test which is currently described as the gold standard. *Methods*. 29 hemato-oncological patients with platelet counts between 30000/*μ*L and 70000/*μ*L were recruited for this pilot study. Complications like petechial bleedings, muscle convulsion, and pain were assessed using the Brief Pain Inventory before and 48 hours after the MVC test, which was performed unidirectionally for the quadriceps muscle. *Results*. We did not detect any statistically significant test-related exacerbations or pain development. *Discussion*. MVC testing seems to be a feasible method to control a resistance training program in hemato-oncological patients. Further studies need to extend their methods and, for example, compare the MVC test with the one repetition maximum test.

## 1. Introduction

Hemato-oncological patients who underwent chemotherapy often show a severe reduction in muscle mass. This reduction may be caused by immobilization, the influence of chemotherapeutic agents, and/or the impact of immunosuppressive therapy consisting of glucocorticoids and cyclosporine and counteracting the graft-versus-host-disease [1–9]. The decrease of muscle mass leads to a loss of physical performance and is involved in certain cancer and therapy associated phenomena like cancer-related fatigue and cachexia [10, 11]. Especially patients suffering from cachexia have a poor prognosis and a reduced tolerance regarding intensive treatment protocols including radiation and chemotherapeutic agents [12].

The positive influence of different types of exercise, for example, endurance and resistance training, on physical and psychological outcomes in cancer patients has been proven in several studies [13–15]. Yet evidence-based guidelines regarding resistance training frequencies and intensities are still missing. Possibly more valid and practicable assessment methods would help to generate specific recommendations. Although leukemia and lymphoma patients have a comparably long hospitalization phase and therefore experience long periods of immobilization and high muscle atrophy rates, resistance training is rarely recommended [16, 17]. Most authors suggest a moderate endurance exercise program for hemato-oncological patients because of its modest impact on the cardiovascular system. Resistance training in return is often associated with imposing a harmful load [7, 18]. As in patients with heart transplantations, the fear of high blood pressure and hemorrhage due to thrombocytopenia often leads to the decision that resistance training should not be offered to hemato-oncological patients, although they could considerably benefit from it [19]. Resistance training with an intensity of approximately 50–60% of a maximal voluntary contraction (MVC) does not result in high blood pressure values and even has protective effects on the cardiovascular system [20]. An increasing number of studies have demonstrated that resistance training programs with hemato-oncological patients with platelet counts as low as 50000/*μ*L are feasible [10].

The oncology experts of the American college of sports medicine (ACSM) recommend conducting the one repetition maximum (1RPM) test prior to a resistance training program [21]. However, the 1RPM test can only be performed if patients are not in isolation and have the possibility to visit a facility with special equipment (machines, weights, etc.) An alternative and inexpensive assessment tool that is commonly used to control resistance training is an MVC test. An MVC test is a well-established, reliable, and valid instrument, as is the 1RPM test [22, 23].

Since the lower limbs are strongly affected by immobilization-induced atrophy [11], we examined whether an isometric MVC test for the quadriceps muscle is feasible in hemato-oncological patients during thrombocytopenia. 

## 2. Methods

### 2.1. Patients

**Figure 1:**
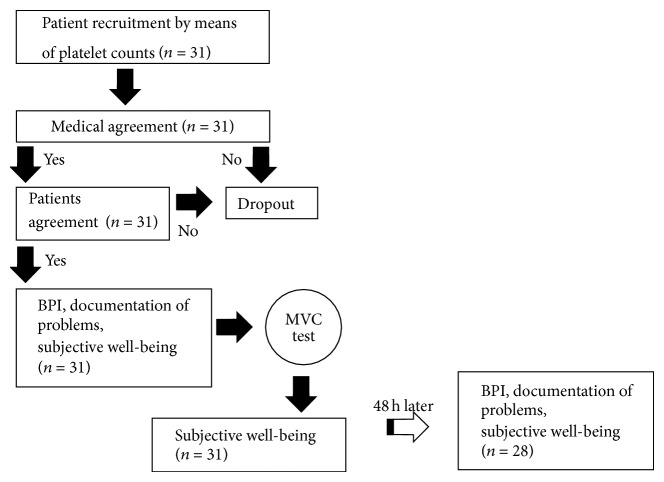
Patient enrolment.

31 hemato-oncological patients with different diagnoses and chemotherapeutic treatment protocols were recruited into this feasibility study during their hospital stay (Figure 1). All patients were older than 18 years and provided a written informed consent prior to the intervention allowing scientific data evaluation according to the standards published by Harriss and Atkinson [24]. Two patients were excluded from the study due to the fact that they were released from the hospital prior to the posttest assessments. This pilot study was approved by the ethics committee of the University of Cologne, Germany (No. 07-241).

Patients with platelet counts above 70000/*μ*L or below 30000/*μ*L as well as patients with hemoglobin values below 8 g/dL were excluded from the intervention. First studies show that physical activity programs are feasible for patients with even lower platelet counts [10]; however, Rogge recommend physical inactivity for these patients [25]. Ongoing chemotherapy, cardiovascular diseases, systolic blood pressure over 150 mmHg, orthopedic handicaps, and hemorrhage were further exclusion criteria (Figure 1). Patient characteristics are listed in Table 1.

### 2.2. MVC Test

The isometric MVC test of the quadriceps muscle was performed unidirectionally with a customary load cell (DigiMax, MechaTronic GmbH, Hamm, Germany). During the test, patients sat on a cot, and their feet did not reach the floor. Their hip and knee joints were positioned in a 90-degree flexion angle. Additionally, all patients were fixed with a hip belt to minimize the influence of the hip muscles. The sensor was fixed underneath the cot, vertical to the movement axis of the shank on the one side and around the patient's ankle on the other side. The lever arm was measured from the gap of the knee joint to the middle of the sensor fixing collar. Prior to the test, the position of the collar was marked with a pen in order to ensure equal conditions for all three trials. All patients were briefed in an equal manner and by the same therapist. As soon as the patients sat in the right position, they were asked to keep their back straight and fold their arms in front of their abdomen. The therapist counted down five seconds and then said “Maximal force now.” At this point of the test, patients were to reach their MVC. During the complete contraction phase, patients were asked to breathe out. This procedure was repeated three times. A one-minute break was taken after every trial.

### 2.3. Documentation of Complications and Exacerbations

Complications and exacerbations were documented before and two days after the MVC test. They were classified into four stages:

 Stage 1: aching muscles and/or hematoma,
 Stage 2: petechiae,
 Stage 3: pain in the limbs or in the trunk,
 Stage 4: disruptions of muscles or tendons.

### 2.4. Brief Pain Inventory (BPI)

In order to investigate the influence of the MVC test on pain intensity (sensory dimension) and degree of impairment of daily living (reactive dimension), the German version of the BPI questionnaire was applied [26]. The BPI was completed directly before and two days after the MVC test.

### 2.5. Subjective State of Well-Being

The subjective state of well-being was assessed by means of an open question before, immediately after, and 48 hours after the MVC test. The investigator asked all patients: “Do you think or feel that the MVC test influenced your well-being?”

### 2.6. Statistical Analyses

Complications and exacerbations were analyzed with the McNemar test. Pain rates were calculated in per cent. Pain intensity as well as pain associated derogation was compared with the Wilcoxon test. The subjective state of well-being was analyzed descriptively. SPSS 20 was used for all statistical analyses. 

## 3. Results

### 3.1. Complications and Exacerbations

Pretest complications were documented in nine patients. These mild complications were associated with convulsions during the night, bone marrow biopsies, and knee problems that afflicted the knee which was not stressed in the MVC test. In the posttest, 2 days after the intervention, the same complications were observed in six patients. Two patients reported exacerbations (Table 2).

Stage one complications (aching muscles) were documented in one individual before and in two individuals after the MVC test. One of these patients had aching muscles in both legs, although the MVC test was performed unidirectionally. Stage two complications were observed in two patients. These complications had the same dimension in the pre- and the posttest. No petechiae could be discovered in the area where the collar was fixed. Stage three complications were reported by six patients before and four patients after the MVC test. None of these patients associated the complications with the intervention. Stage four complications and exacerbations were neither observed in the pre- nor in the posttest.

### 3.2. Pain

58 BPI questionnaires were completed by 29 patients. In the Pretest assessment seven patients (24.14%) declared that they experienced pain during the last 24 hours. Only four of these seven patients report pain in the posttest (13.79%). 22 patients did not experienced any pain before or after the test. Regarding the sensory dimension of the BPI the difference between the pre- and the posttest was not significant (*P* = 0.063). The reactive dimension showed no difference either (*P* = 0.23).

Of the seven individuals with pain, one patient indicated mild muscle convulsion and pain in thigh and shank in the BPI Pretest. However, no exacerbation was documented in the BPI posttest. Two of the patients reported that their pain increased. In one of the individuals the pain was located in the iliac crest and was associated with a bone marrow biopsy. The degree of pain showed a mild increase (Pretest: 3.25, Posttest: 3.75) whereas the pain induced impairment increased from 0.43 to 5.0 in this individual. The second patient, that reported exacerbations regarding pain intensity (Pretest: 3.5, Posttest: 3.75) and impairment (Pretest: 4.86, Posttest: 5.43), had unidentified complications in the untested knee. Exacerbations were only mild in both outcomes.

### 3.3. Subjective State of Well-Being

Patients reported that the MVC test did not influence their subjective state of well-being. 

## 4. Discussion

Every year thousands of cancer patients take part in exercise programs during their hospitalization and rehabilitation phase. Most of these patients cannot benefit properly from resistance training due to one of the following points. Firstly, (i) although there is an increasing number of studies dealing with resistance training in cancer patients, therapists often have a gap of knowledge regarding the feasibility and the positive effects of such an intervention. It is therefore necessary to include this knowledge into education programs for nurses, physiotherapists, and exercise therapists. (ii) Secondly, the technical and financial resources vary between the different facilities. 

In order to optimize the outcome of an exercise intervention, a practicable and inexpensive assessment which can be used in all kinds of settings is indispensable. To the best of our knowledge, an assessment for resistance training which is applicable for patients in isolation and comparable to the VO_2_ max test or the WHO test in endurance exercise programs is still missing [21]. This leads to the circumstance that a great number of hemato-oncological patients may not benefit from the positive effects of such a resistance training program. Furthermore, studies have to deal with small sample sizes since many patients have low platelet counts during their therapy. This also affects other groups of cancer patients during thrombocytopenia.

In order to develop and evaluate an individual resistance training program for hemato-oncological patients especially during isolation, it is essential to standardize a strength test. In this pilot study, we were able to demonstrate that an MVC test is save and feasible in our collective.

Existing complications only increased in one patient and in this case were not associated with the test. Taken together, statistical analyses did not show any difference in complications between the pre- and the posttest. Furthermore, the results suggest that relative pain rate, pain intensity, and the influence of pain on daily activities are not affected by the MVC test. Finally, patients reported that their subjective state of well-being was not influenced by the test.

Therefore, the MVC test may serve as a sound basis for a controlled exercise program, especially for patients who are not able to perform a 1RPM test due to isolation or the missing financial, infrastructural potential of the facility. A practicable assessment like the MVC test is necessary to evaluate exercise interventions for cancer patients during isolation. We assume that a hypertrophy training, which involves even smaller loads than those imposed during the MVC test, might be possible in hemato-oncological patients with platelet counts as low as 30000/*μ*L. In contrast to other studies but in accordance with the studies of Elter et al. [10] and Baumann et al. [9, 27], we conducted our research with patients who had platelet counts between 30000/*μ*L and 70000/*μ*L. 

The existing studies suggest that a resistance exercise program for hemato-oncological patients should only be approved if patients' platelet counts exceed 50000/*μ*L [6]. The study group “Physical Activity and Cancer” of the German cancer society recommends resistance training if platelet counts exceed 30000/*μ*L [25]. This recommendation is based on an evidence level grade IV. 

Callow et al. were able to show that hemorrhage risk is not proportional to platelet counts [28]. Based on this study, Baumann et al. [9] found that an endurance exercise program is feasible for hemato-oncological patients who have thrombocytopenia values between 8000/*μ*L and 27000/*μ*L. First nonsystematic experiences of our study group indicate that a MVC test seems to be feasible at platelet counts as low as 20000/*μ*L. During these tests systolic blood pressure was lower than 150 mmHg. Additionally, a combined submaximal endurance and resistance training could be conducted when platelet counts were only 10000/*μ*L [9, 27].

We intentionally decided to measure the force using a simple sensor because this inexpensive and practicable instrument can also be applied outside of the professional physiotherapeutic setting. A 1RPM test on the other hand can only be conducted if different kinds of weights or exercise machines are available. Therapists can easily evaluate the MVC force in a patient's hospital room. This can be an advantage regarding hemato-oncological patients in isolation. Nevertheless, an MVC test should always be conducted by a professional in order to ensure that forced respiration and fitful movements are avoided during the test. Aside from the 1RPM test, the rating of perceived exertion (RPE) scale can also be used to control resistance training. However, to optimize the effect this subjective instrument should only be used in combination with an objective instrument like an MVC test. 

The results of the present pilot study are limited. Further research in this field should involve larger sample sizes and compare the described method directly with the gold standard. Regarding the evaluation of an MVC test, further studies should also include other muscle groups aside from the quadriceps. Potential outcomes of interest could be the serum creatine kinase to evaluate the rate of muscle damage and the international normalized ratio (INR) to monitor the clotting tendency of the patient's blood. Finally, pain should also be assessed immediately after the MVC test in order to investigate possible negative short term effects.

## 5. Conclusion

In summary, we postulate that an isometric MVC test is feasible in hemato-oncological patients with platelet counts above 30000/*μ*L and may serve as a basis for a controlled resistance training for the lower limbs. Besides an individualized endurance training, resistance training will play a key role in maintaining the physical performance of cancer patients, especially during the medical therapy. By involving an isometric MVC test, nurses, therapists, and doctors are able to recommend specific exercise programs without having to fear any possible overload for their patients. Further research in this field is necessary to underline and expand the presented findings.

## Figures and Tables

**Table 1:** Patient characteristics.

Disease	Number of patients
AML	8
ALL	5
HL	6
NHL	8
CML	1
MM	1
Total	29

Platelet counts ×10^3^/*μ*L	
Mean = 47.34	
SD = 11.44	
Leukocytes ×10^3^/µL	
Mean = 5.43	
SD = 7.24	
Hemoglobin g/dL	
Mean = 10.49 SD = 1.1	
Sex	
m = 12	
f = 17	
Age	
Mean = 51.83 SD = 14.25	

SD: standard deviation; m: male; f: female.

**Table 2:** Results of the McNemar test and absolute number of different complications in the pre- and the posttest.

Complications	Yes	No	
Pretest (45%)	9	20	29
Posttest (40%)	8	21	29
	17	41	*P* = 1,0

Complications	Stage 1	Stage 2	Stage 3

Pre test	1	2	6
Posttest	2	2	4
